# Incidence Survey of Acute Otitis Media in Children in Sado Island, Japan—Sado Otitis Media Study (SADOMS)

**DOI:** 10.1371/journal.pone.0068711

**Published:** 2013-07-02

**Authors:** Taketo Otsuka, Osamu Kitami, Kouji Kondo, Hisayuki Ota, Shinsuke Oshima, Akio Tsuchiya, Takatoshi Shirai, Koyata Fujii, Michihide Nakamure, Yasuhiro Shoji, Hisano Nakamura, Yasuko Masuda, Kenichi Komiyama, Kazunaga Yoshida, Yukio Ishikawa, Atsushi Iwaya, Sugata Takahashi, Minoru Okazaki, Muneki Hotomi, Noboru Yamanaka

**Affiliations:** 1 Department of Pediatrics, Sado General Hospital, Sado, Niigata, Japan; 2 Shiseido Otolaryngology Clinic, Sado, Niigata, Japan; 3 Kondo Clinic, Sado, Niigata, Japan; 4 Department of Otolaryngology, Sado General Hospital, Sado, Niigata, Japan; 5 Department of Pediatrics, Ryotsu Hospital, Sado, Niigata, Japan; 6 Department of Clinical Laboratory, Sado General Hospital, Sado, Niigata, Japan; 7 Department of Otolaryngology, Niigata University, Niigata-shi, Niigata, Japan; 8 Department of Otolaryngology-Head and Neck Surgery, Wakayama Medical University, Wakayama-shi, Wakayama, Japan; The University of Tokyo, Japan

## Abstract

**Background:**

Acute otitis media (AOM) is one of the most common forms of bacterial infection and cause for clinic visits in children. The incidence of AOM was 0.9–1.2 episodes per person-year during the first 2 years of life in previous reports conducted before 2000. The aim of this study was to 1) evaluate the latest AOM incidence in pediatric outpatients and 2) identify the bacterial pathogens from these patients and ascertain their serotypes and resistance.

**Methods:**

The study was conducted in a closed population, involving all pediatricians and otolaryngologists in Sado Island allowing accurate determination of AOM incidence. In each month, one week was assigned as “surveillance week”, and all outpatients with acute illness aged 0–18 years examined during the surveillance weeks were enrolled. AOM was diagnosed on the basis of otoscopic findings and clinical symptoms were recorded. Specimens were collected from the nasopharynx or middle ear cavity of AOM patients and examined for bacteria. Antimicrobial susceptibilities, serotypes, and molecular typing for resistance were determined among *Streptococcus pneumoniae* and *Haemophilus influenzae.*

**Results:**

In total, 8,283 clinic visits were conducted, and 354 episodes (4.3%, 95% CI: 3.9–4.7%) among 312 children were diagnosed as AOM. The incidence of AOM was highest in children of 1 year of age (0.54 episodes/child/year, 95% CI: 0.44–0.64). Serotype coverage of 7- and 13-valent pneumococcal conjugate vaccines in this study were 38.0% (95% CI: 29.3–47.3) and 62.8% (95% CI: 53.6–71.4), respectively. Of 122 *H.influenzae* isolates available for typing, 120 were nontypeable and 2 were type b. A high proportion of *S. pneumoniae* isolates (46%) showed resistance to penicillin. Approximately half of *H. influenzae* isolates had genetic markers for beta-lactamase-negative ampicillin-resistance.

**Conclusions:**

Approximately 4–5% of pediatric outpatients, even without AOM-related symptoms, had AOM in our study. Pediatricians as well as otolaryngologists should check the tympanic membrane findings of all pediatric outpatients.

## Introduction

Acute otitis media (AOM) is one of the most common forms of bacterial infection and cause for clinic visits in children, with 70% of children experiencing at least one episode by age 3 [1], [2]. The incidence of AOM were reported as 1.1–1.2 episodes per person-year during the first 2 years of life in the United States (conducted between 1975 and 1984) [2], 1.16–1.24 episodes per person-year from 6.5 to 24 months of age in Finland (conducted between 1995 and 1999) [3], and 0.9 episodes per person-year during the first 2 years of life in Bangladesh (conducted between 1993 and 1996) [4]. However, these rates were estimated during the 20^th^ century, and there have been time-dependent changes in the current conditions surrounding children, such as vaccines, antimicrobials, day-care use, living environment, and allergies.

*Streptococcus pneumoniae*, nontypeable *Haemophilus influenzae* (NTHi), and *Moraxella catarrhalis*, which are members of the commensal flora of the nasopharynx, are the 3 leading causes of AOM. The frequency of nasopharyngeal colonization by these pathogenic bacteria is strongly associated with AOM [5], [6].

There have been few studies on the epidemiology (incidence, severity, and etiology) of AOM in Japan. According to the subcommittee regarding AOM guidelines, the exact frequency of AOM occurrence in Japan is unknown [7]. The aim of this study was to 1) evaluate AOM proportion in pediatric outpatients and incidence in Sado Island, Niigata, Japan and 2) identify the bacterial pathogens from these patients and ascertain their resistance.

## Methods

### Study Area and Population

The study, Sado Otitis Media Study (SADOMS), was conducted in Sado Island, which lies 60 km off the Japanese mainland, between January 2010 and December 2011. In Japan, *H. influenzae* type b (Hib) conjugate vaccine and 7-valent pneumococcal conjugate vaccine (PCV7) were introduced for voluntary immunization in December 2008 and February 2010, respectively. However, they have not been implemented as part of a routine vaccination program in Japan, and they have only been provided free of charge since January 2011 (See Table 1). SADOMS phase I was undertaken between January 2010 and December 2011 (the PCV7-introductory era), phase II will be undertaken between January 2012 and December 2013 (early phase of the post-PCV7 era), and phase III will be undertaken between January 2014 and December 2015 (late phase of the post-PCV7 era). At the time when the study was started (January 2010), the population of the island was 64,120, with 9,388 (14.6%) aged 0–18 years. Children aged less than five years constituted 3.4% (2,167) of the total population. Here, we report on SADOMS phase I.

**Table 1:** Incidence and severity of AOM by age, and vaccination coverage during 2 years surveillance.

					AOM yearly incidence (episodes/child/year)				Vaccination coverage (%)					
									PCV7				Hib vaccine	
Age (years)	Population(person)*	Clinic visit(per 2 years)	AOM episodes(per 2 years)	Overall	(95% CI)	Severe	Moderate	Mild	Entire Sado population**	Among AOM patients	*p*-value	Entire Sado population **	Among AOM patients	*p*-value
														
0	400	893	41	0.22	0.16–0.30	0.10	0.10	0.02	49.3%	26.8%	<0.01	50.0%	29.3%	0.01
1	420	1,289	104	0.54	0.44–0.64	0.21	0.23	0.10	23.7%	13.5%	0.03	23.2%	13.5%	0.04
2	428	1,117	68	0.34	0.27–0.43	0.06	0.13	0.15	22.0%	13.2%	0.14	19.9%	14.7%	0.32
3	443	893	43	0.21	0.15–0.28	0.02	0.06	0.13	18.6%	16.3%	0.86	19.2%	18.6%	0.91
4	449	679	29	0.14	0.09–0.20	0.02	0.04	0.08	20.7%	3.4%	0.04	17.0%	3.4%	0.10
5	421	628	18	0.09	0.06–0.15	0.02	0.04	0.03	N.A.	0.0%	N.A.	N.A.	0.0%	N.A.
6	389	457	10	0.06	0.03–0.10	0.01	0.01	0.04	N.A.	0.0%	N.A.	N.A.	0.0%	N.A.
7–18	6324	2327	41	0.01	0.01–0.02	0.00	0.00	0.01	N.A.	0.0%	N.A.	N.A.	0.0%	N.A.

* Average of January 2010 and January 2011 in Sado Island.

** Vaccination coverage in Sado Island on November 2011.

AOM, acute otitis media; PCV7, 7-valent pneumococcal conjugate vaccine; Hib, *H. influenzae* type b; N.A., not available.

### Surveillance

The second week of each month was assigned as a surveillance week for observational surveillance. When the second week had a national holiday, the first or third week was assigned as a surveillance week. All outpatients with acute illness aged 0–18 years examined during the surveillance weeks were enrolled in this study. Acute illness was defined as the presence of one or more of the following symptoms: fever, cough, rhinorrhea, fatigue, nausea, vomiting, diarrhea, otalgia, exanthema, and irritability. Because symptoms of respiratory tract infections among children can be non-specific, our definition of acute illness could include non-respiratory tract infections, such as gastrointestinal infection, urinary tract infection, and acute phase of chronic diseases. Children visiting a doctor for health checkups, vaccination, or treatment of chronic diseases were excluded from this study. No outpatient could be enrolled twice in the same surveillance week, while each outpatient could be enrolled every surveillance week. Each pediatric outpatient was seen by a doctor belonging to the SADOMS study group in Sado Island. The group included all pediatricians (6 doctors) and all otolaryngologists (3 doctors) in Sado Island. We covered 97.8% (10,310/10,538 visits) of the pediatric patients who visited hospitals/clinics due to acute illness during their first 3 years in Sado Island [8]. Although some outpatients >3 years old are not followed by the study group doctors, the referral system in Sado Island functions such that children with AOM regardless of age are referred to the 6 pediatricians or the 3 otolaryngologists. Therefore, it is likely that almost all AOM patients were enrolled in this study.

AOM was diagnosed on the basis of tympanic membrane findings; 1) the presence of middle ear effusion, and 2) signs of middle-ear inflammation including redness and bulging [9], [10]. Also, we recorded a history of acute onset of signs and symptoms, such as fever, irritability, and otalgia (but, they are non-specific). AOM severity was diagnosed as mild, moderate, or severe, based on the scoring system included in the 2009 Japan AOM clinical guidelines [7]. In addition, an outpatient who was enrolled in the surveillance was counted as having AOM, if he/she was diagnosed with AOM within 10 days from his/her clinic visit. The reason is that most AOM cases cannot be diagnosed at first visit soon after developing the first symptoms of acute illness.

A tympanogram is a useful adjunct for the diagnosis of AOM in doubtful cases and has a 90% sensitivity to predict middle ear effusion [11]. However, a tympanogram is not always feasible in pediatric departments in Japan. Thus, our study pediatricians relied instead on otoscopic findings. To standardize the otoscopic skills, all 10 doctors who are experienced clinicians, were trained again prior to the study and used the same atlases/texts for AOM diagnosis based on the guidelines [9]. As part of our study protocol, pediatricians consulted otolaryngologists when the pediatrician’s diagnosis of AOM was doubtful. The indications for a myringotomy were 1) tympanic membrane bulging or middle ear fluid, and 2) severe ear pain. An otolaryngologist determined the necessity for myringotomy based on AOM severity and patient’s age.

The subjects’ medical information was obtained from their patient’s case records and by questionnaires. Information included i) the past history of AOM, and ii) vaccination status with Hib and PCV7 vaccines.

The AOM proportion for all outpatients with acute illness was calculated in each surveillance week. In addition, clinic visit-based incidence of AOM by age (episodes/child/year) was calculated from the census of Sado city. The number of AOM patients during the study period was estimated by multiplying the observed episodes by 104/24 (total number of weeks in 2 years study period/number of weeks of observation). Dividing by 2, we obtained the annual number of AOM episodes. Then, dividing by each age population, the clinic visit-based incidence of AOM (episodes/child/year) was calculated. We assumed that each “surveillance week” represented the month. Otitis prone was defined as 3 separate episodes in 6 months or 4 episodes in 1 year.

### Bacterial Cultures

Specimens were collected from the nasopharynx or middle ear cavity and examined for bacteria. The middle ear fluid samples were obtained by myringotomy or from ear discharge.

Except for myringotomy samples, these cultures were obtained from AOM patients using a sterile cotton-tipped aluminum swab (Seed Swab No. 2; Eiken Chemical Co. Ltd., Tokyo, Japan). Swabs were cultured immediately on chocolate and sheep blood agar plates and incubated at 36°C in 5% vol/vol CO_2_ for 18–24 hours. *S. pneumoniae* strains were identified by colony morphology, susceptibility to optochin, and bile solubility. *H. influenzae* strains were identified by colony morphology and the use of an automated diagnostic system (HNID panel with the MicroScan Walk Away-96; Dade Behring Inc., West Sacramento, CA). Then, the pathogens were confirmed as *H. influenzae* isolates by a PCR method identifying the 16S ribosomal DNA and IgA protease gene [12]. *M. catarrhalis* strains were identified by colony morphology and the presence of butyrate esterase. The predominant colony type on each plate was selected for this study.

All isolates of *S. pneumoniae* and *H. influenzae* were stored at −80°C until further analysis.

### Antibiotic Susceptibility Testing

Antimicrobial susceptibilities were determined using commercially available MIC panels (MICroFAST 3J for *S. pneumoniae* and 4J for *H. influenzae;* Dade Behring Inc., Deerfield, IL, USA). MICs were interpreted as susceptible, intermediate, or resistant according to the guidelines set by the Clinical and Laboratory Standards Institute (CLSI) [13]. The following antimicrobial agents were tested: penicillin G (PEN), erythromycin, and clindamycin for *S. pneumoniae*; and ampicillin (AMP) and amoxicillin-clavulanic acid (AMC) for *H. influenzae*.

### Serotyping

The isolates of *H. influenzae* and *S. pneumoniae* were serotyped with a commercially available slide agglutination kit (Denka Seiken Co., Tokyo, Japan), and by the capsular swelling method (Quellung reaction) using commercially available antisera (Statens Serum Institutes, Copenhagen, Denmark). Isolates that showed negative reactions for all pooled sera were considered to be nontypeable.

### Molecular Typing for Resistance

Molecular typing for resistance was determined by PCR. Bacterial DNA was extracted from the isolates using a High Pure PCR Template Preparation Kit (Roche Diagnostics GmbH, Mannheim, Germany) according to the manufacturer’s instructions. Macrolide-lincosamide-streptogramin B (MLS_B_) resistance genes (*ermB* and *mefA/E*), and mutations for penicillin resistance (*pbp 1a, pbp 2x,* and *pbp 2b* mutations) in *S. pneumoniae* were identified by PCR [14]–[16]. *S. pneumoniae* isolates were classified into 3 genotypes by the presence of *pbp 1a, pbp 2x,* and *pbp 2b* mutations: penicillin susceptible *S. pneumoniae* (gPSSP, no mutation), penicillin intermediate *S. pneumoniae* (gPISP, with 1 or 2 mutations), and penicillin resistant *S. pneumoniae* (gPRSP, with all mutations).

The beta-lactamase gene (*bla*) and *ftsI* mutations in *H. influenzae* were identified by PCR [17]. *H. influenzae* isolates were classified into 4 genotypes on the basis of the PCR-based genotyping: beta-lactamase-negative AMP-susceptible isolates, beta-lactamase-negative AMP-resistant (gBLNAR, and subdivided I/II and III) isolates, beta-lactamase-positive AMP-resistant isolates, and beta-lactamase-positive AMC-resistant (subdivided I/II and III) isolates [17].

### Data Analysis

The vaccination coverage by age among the entire Sado population and AOM patients, and the rates of bacterial carriage for each level of severity were compared. These analyses were performed by chi-square test and Fisher’s exact test. Adjusted standardized residuals were calculated, when the rate of bacterial carriage was significantly different between groups based on AOM severity. *P*-values <0.05 were considered significant. All statistical calculations were performed with PASW Statistics 18.0 for Windows (SPSS Inc., Chicago, IL, USA).

### Ethics Statement

Because this study was an observational study and sampling of bacteria was a routine work in AOM treatment, verbal informed consent was obtained from the guardians of all children with AOM when the questionnaire was filled out. The bacteria samples and data sheets were anonymized. This study protocol including consent procedure was approved by the Ethics Committee in Sado General Hospital.

## Results

**Figure 1:**
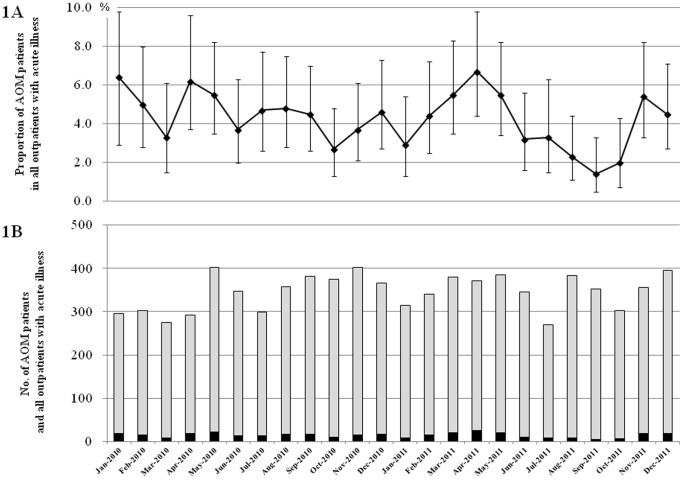
Pediatric patients with acute otitis media in Sado Island by surveillance week 1A. The proportion of acute otitis media patients with 95% CI among all outpatients showing acute illness. 1B. The black bar and gray bar indicate the number of acute otitis media patients and all outpatients with acute illness, respectively. AOM, acute otitis media.

In total, 8,283 (male 4,432 and female 3,851) clinic visits for acute illness were conducted, at which 354 AOM episodes (4.3%, 95% CI: 3.9–4.7%) were detected (Figure 1). Considering recurrent episodes, 312 children were diagnosed as AOM at least 1 time during study period. AOM proportion in outpatients were 5.5% (95% CI: 4.9–6.1%) among patients aged 0–5 years, and 1.8% (95% CI: 1.4–2.4%) among patients aged 6–18 years. Of 8,283 outpatients, pediatricians consulted otolaryngologists for <100 children with diagnosed AOM and doubtful AOM. During this study, the number of clinic visits in each surveillance week ranged from 269 (in July 2011) to 402 (in May 2010 and November 2010). The number and proportion of AOM episodes among outpatients ranged from 5 (1.4%) in September 2011 to 25 (6.7%) in April 2011. The analysis for seasonality was not done, because we judged that 2 years surveillance was not enough for such an analysis.

Of 312 children with AOM, 162 (51.9%) were the first episode and 146 (46.8%) had recurrent episodes, including 21 (6.7%) otitis prone children, at the time they were diagnosed with AOM in this study. Past AOM histories in 4 AOM patients were not available. The clinic visit-based incidence of AOM was highest in children of 1 year of age (0.54 episodes/child/year, 95% CI: 0.44–0.64) and tended to decrease in older children (Table 1). Seventy and 143 were diagnosed as severe and non-severe AOM, respectively, among AOM patients aged 0–2 years. On the other hand, 16 and 125 were diagnosed as severe and non-severe AOM, respectively, among AOM patients aged >3 years. Younger children were more likely to be diagnosed with severe AOM (*p*<0.001).

Of 354 episodes, 310 cultures from nasopharyngeal (283 specimens; 91.3%) and middle ear (27 specimens; 8.7%) cavities were obtained. Of 283 specimens from nasopharynx, 143 (50.5%) were positive for *S. pneumoniae*, 130 (45.9%) were positive for *H. influenzae*, and 127 (44.9%) were positive for *M. catarrhalis*. On the other hand, among 27 middle ear fluid samples, 6 (22%) were positive for *S. pneumoniae*, 10 (37%) were positive for *H. influenzae*, and 3 (11%) were positive for *M. catarrhalis*. (Table 2). We obtained a relatively small number of middle ear fluid samples, because the clinical efficacy of myringotomy is still controversial [7].

**Table 2:** Severity and bacterial pathogens in acute otitis media.

		Number of bacterial pathogens (%)											
		Nasopharynx					Middle ear fluid						
Severity		*S.pneumoniae*	*H.influenzae*	*M. catarrhalis*	Either^a^	Combined^b^			*S.pneumoniae*	*H.influenzae*	*M. catarrhalis*	Either^a^	Combined^b^
Total	(n = 283)	143(50.5%)	130(45.9%)	127(44.9%)	245(86.6%)	132(46.6%)		(n = 27)	6(22%)	10(37%)	3(11%)	15(56%)	4(15%)
Mild	(n = 104)	53(51%)	39(38%)	43(41%)	86(83%)	40(38%)		(n = 5)	1(20%)	1(20%)	0(0%)	1(20%)	1(20%)
Moderate	(n = 113)	61(54%)	62(55%)	45(40%)	99(88%)	60(53%)		(n = 5)	2(40%)	2(40%)	1(20%)	4(80%)	1(20%)
Severe	(n = 66)	29(44%)	29(44%)	39(59%)	60(91%)	32(48%)		(n = 17)	3(18%)	7(41%)	2(12%)	10(59%)	2(12%)
*p-*value^c^		0.43	0.03	0.03	0.28	0.09			N.A.	N.A.	N.A.	N.A.	N.A.

a at least 1 of these pathogens was isolated.

b at least 2 of these pathogens were isolated.

c Comparing with mild, moderate, and severe groups.

N.A., not analyzed.

Most patients with AOM (83.9%, 260/310) had at least 1 AOM pathogen and nearly half of the patients (43.9%, 136/310) had 2 or more AOM pathogens. As shown in Table 2, there were significant differences in the carriage rates of these nasopharyngeal pathogens between severe, moderate, and mild AOM. *H. influenzae* isolates were more likely to be detected in moderate AOM cases, while *M. catarrhalis* were more likely to be detected in severe cases.

**Figure 2:**
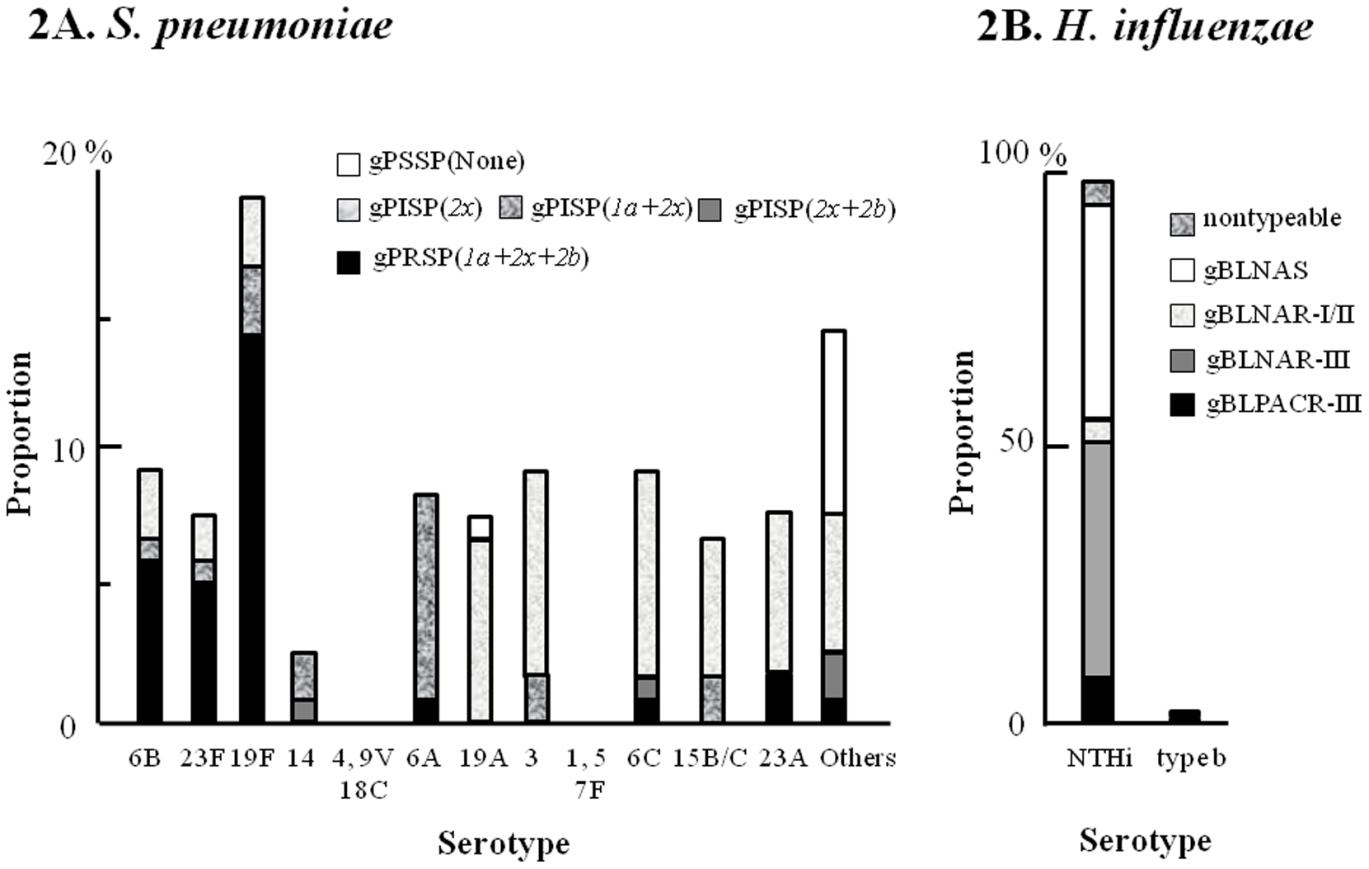
Proportion of serotypes and resistance genes in acute otitis media patients 2A. *S. pneumoniae.* Serotype coverage of 7- and 13-valent pneumococcal conjugate vaccine in this study were 38.0% (95% CI: 29.3–47.3) and 62.8% (95% CI: 53.6–71.4%), respectively. 2B. *H. influenzae.* Of 122* H. influenzae* available for typing, 120 were NTHi and 2 were Hib. Resistance genotyping showed that 9% were gBLPACR-III, 43% were gBLNAR-III, 4% were gBLNAR-I/II, 40% were gBLNAS, and 4% were nontypeable. gPSSP, genetically penicillin susceptible *S. pneumoniae*; gPISP, genetically penicillin intermediate *S. pneumoniae*; gPRSP, genetically penicillin resistant *S. pneumoniae*; gBLNAS, genetically beta-lactamase-negative ampicillin-susceptible isolates; gBLNAR, genetically beta-lactamase-negative ampicillin-resistant isolates; gBLPACR, genetically beta-lactamase-positive ampicillin-resistant isolates; NTHi, nontypeable *H. influenzae.*

Serotype distribution of *S. pneumoniae* and their resistant genes in AOM patients is shown in Figure 2A. Of 149 *S. pneumoniae* isolates, 121 were available for typing. Frequently isolated *S. pneumoniae* serotypes included 19F (19%, 23/121), 3 (9%, 11/121), 6C (9%, 11/121), 6B (9%, 11/121), 6A (8%, 10/121), 19A (7%, 9/121), 23F (7%, 9/121), and 23A (7%, 9/121). Serotype coverage of PCV7 and 13-valent PCV (PCV13) in this study were 38.0% (95% CI: 29.3–47.3) and 62.8% (95% CI: 53.6–71.4), respectively. Genetic classification of resistance among *S. pneumoniae* isolates revealed that 29% were gPRSP (*1a+2x+2b*), 17% were gPISP (*1a+2x*), 9% were gPISP (*2x+2b*), 38% were gPISP (*2x*), and 7% were gPSSP (Table 3A). Antimicrobial susceptibilities testing showed that 46% (56/121) of the *S. pneumoniae* isolates were resistant to PEN. On the other hand, 15% of the *S. pneumoniae* isolates had both *ermB* and *mefA/E* genes, 50% had *ermB* gene, 21% had *mefA/E* gene, and 14% had neither *ermB* nor *mefA/E* genes. These MLS_B_ resistance genes were consistent with the antimicrobial susceptibility testing results for those isolates with 3 exceptions. With regard to *pbp* mutations and MLS_B_ resistance genes, almost all gPRSP isolates carried MLS_B_ resistance genes (both or either), while 87% of gPISP isolates and 44% of gPSSP isolates carried MLS_B_ resistance genes (both or either), respectively (Table 3A).

**Table 3:** PCR-based genotyping and susceptibility of acute otitis media isolates.

PCR-based genotype			Susceptibility to Penicillin G			MLS_B_ genes			
	n	(%)	Susceptible	Intermediate	Resistance	*mefA/E+ermB*	*mefA/E*	*ermB*	none
gPSSP (none)	9	(7%)	9	0	0	1	1	2	5
gPISP (2x)	46	(38%)	43	3	0	10	3	32	1
gPISP (1a+2x)	20	(17%)	10	10	0	3	2	7	8
gPISP (2x+2b)	11	(9%)	2	9	0	0	3	7	1
gPRSP (1a+2x+2b)	35	(29%)	1	24	10	4	16	13	2
Total	121	(100%)	65 (54%)	46 (38%)	10 (8%)	18 (15%)	25 (21%)	61 (50%)	17 (14%)

PCR-based genotype			Susceptibility to Ampicillin		
	n	(%)	Susceptible	Intermediate	Resistance
gBLNAS	49	(40%)	48	1	0
gBLNAR-I/II	5	(4%)	3	0	2
gBLNAR-III	52	(43%)	20	15	17
gBLPACR-III	11	(9%)	0	0	11
Others*	5	(4%)	4	0	1
Total	122	(100%)	75 (62%)	16 (13%)	31 (25%)

gPSSP, genetically penicillin susceptible *S. pneumoniae*; gPISP, genetically penicillin intermediate *S. pneumoniae*; gPRSP, genetically penicillin resistant *S. pneumoniae*;

MLS_B_, Macrolide-lincosamide-streptogramin B.

*S. pneumoniae.*

Serotype distribution of *H. influenzae* isolates and their resistance genes in AOM patients is shown in Figure 2B and Table 3B. Of 140 *H. influenzae* isolates, 122 were available for typing, with 120 NTHi and 2 Hib. Genetic classification of resistance among the *H. influenzae* isolates showed that 9% were genetically beta-lactamase-positive AMC-resistant-III isolates, 43% were gBLNAR-III, 4% were gBLNAR-I/II, 40% were genetically beta-lactamase-negative AMP-susceptible isolates, and 4% were nontypeable (Table 3B). Antimicrobial susceptibility testing revealed that 38% (47/122) and 9% (11/122) of the *H. influenzae* isolates were resistant to AMP and AMC, respectively (Table 3B). There was a difference in AMP resistance rate between genotype (56%) and phenotype (38%) among *H. influenzae* isolates. Similarly, there was a difference in PEN resistance rate between genotype (gPRSP+gPISP; 93%) and phenotype (46%) among *S. pneumoniae* isolates.

## Discussion

We conducted a hospital-based AOM surveillance which included almost all AOM cases in Sado Island. We observed that 4.3% (95% CI: 3.9–4.7) of pediatric outpatients (including those with and without the AOM-related symptoms mentioned above) had AOM in our study, indicating that pediatricians should check the tympanic membrane findings of all pediatric outpatients regardless of the presence of symptoms. A history of acute onset of signs and symptoms is controversial for diagnosis of AOM [9], [10]. Shaikh, *et al*. suggest that tympanic membrane bulging is sufficient to diagnose AOM [10].

In addition, we report the clinic visit-based incidence of AOM in an area with limited movement of people (particularly of children) to and from other areas. AOM incidence was highest in children of 1 year of age 0.54 episodes/child/year (95% CI: 0.44–0.64) and tended to decrease in older children. A limitation of our study design is that children with AOM who did not visit hospitals/clinics would be missed. However, our main purpose was to identify the incidence of AOM in all outpatients. It is important for pediatricians to know the AOM frequency and to pay attention to AOM patients in the actual exam room.

We previously reported the serotype distribution of *S. pneumoniae* isolates and their resistance in colonization [8]. In this previous study, the most common serotypes among *S. pneumoniae* isolates were 6B (17.3%), 23F (12.6%), 19F (12.2%), 6C (10.6%), and 6A (8.9%). The serotype distribution of *S. pneumoniae* in isolates from AOM-positive patients differed in comparison to that of healthy colonization, with serotype 3 (1.8% in healthy colonization and 9% in AOM-positive patients) and 19A (2.8% and 7%) increased in isolates from AOM-positive patients, while 6B (17.3% and 9%) and 23F (12.6% and 7%) decreased. PCV13, which includes serotypes 3 and 19A, could prevent the infection and thus AOM.

AOM patients aged 0–1 year showed lower vaccination coverage for PCV7, compared to all children aged 0–1 year in Sado Island (Table 1). A possible explanation is that these children without PCV7 vaccination were more likely to develop AOM. However, only 38.0% of pneumococcal isolates were serotypes that are included in PCV7 in this study. Therefore, we speculate that missing vaccination opportunities caused by illness, such as recurrent infections including AOM, is also an important factor. The observation that younger AOM children had the low coverage of Hib vaccination provides support for the latter explanation, because Hib vaccination has no (or very little) effect to prevent AOM.

Some of the limitations in this study include subject enrollment, sample isolation, and the number of isolates available for genetic characterization. During the time that we enrolled 269–402 patients with acute illness, over 300 patients also visited our hospitals/clinics with chronic diseases, for vaccination, and for follow-up examination in each surveillance week. We decided to use “surveillance week” system because the trends of infectious diseases may gradually change in Sado Island. Over 90% of the cultures evaluated in this study were isolated from the nasopharynx, which included only colonization pathogens. Thus, we could not determine whether these pathogens were causative agents or not. However, there are differences in serotype proportion of *S. pneumoniae* between healthy children and AOM patients.

Approximately 4–5% of pediatric outpatients, even without AOM-related symptoms, had AOM in our study. Pediatricians as well as otolaryngologists should check the tympanic membrane findings of all pediatric outpatients. Pediatric AOM caused by antimicrobial resistant *S. pneumoniae* and *H. influenzae* are still a major concern in Japan. PCV13 as well as vaccines for NTHi could prevent the infection and thus AOM.
